# Caspase-3 Inhibition Attenuates the Cytopathic Effects of EV71 Infection

**DOI:** 10.3389/fmicb.2018.00817

**Published:** 2018-04-26

**Authors:** Fengmei Song, Xiaoyan Yu, Ting Zhong, Zengyan Wang, Xiangling Meng, Zhaolong Li, Shuxia Zhang, Wenbo Huo, Xin Liu, Yahong Zhang, Wenyan Zhang, Jinghua Yu

**Affiliations:** ^1^Institute of Virology and AIDS Research, The First Hospital of Jilin University, Jilin University, Changchun, China; ^2^Department of Experimental Pharmacology and Toxicology, School of Pharmacy, Jilin University, Changchun, China; ^3^College of Pharmacy, Central South University, Changsha, China; ^4^Department of Internal Medicine, The First Hospital of Jilin University, Jilin University, Changchun, China; ^5^Key Laboratory of Natural Medicines and Immunotechnology of Henan Province, Henan University, Kaifeng, China

**Keywords:** EV71, caspase-3, viral production, non-structural protein 3C, host–pathogen interaction

## Abstract

Previous studies demonstrate that human enterovirus 71 (EV71), a primary causative agent for hand, foot, and mouth disease, activates caspase-3 through the non-structural viral 3C protein to induce host cell apoptosis; however, until now it was unclear how 3C activates caspase-3 and how caspase-3 activation affects viral production. Our results demonstrate that 3C binds caspase-8 and caspase-9 but does not directly bind caspase-3 to activate them, and that the proteolytic activity of 3C is required by the activation of caspase-8, caspase-9, and caspase-3. Inhibition of caspase-3 activity attenuates apoptosis in 3C-transfected cells. Furthermore, caspase-3 inhibitor protects host cells from the cytopathic effect of EV71 infection and prevents cell cycle arrest, which is known to be favored for EV71 viral replication. Inhibition of caspase-3 activity decreases EV71 viral protein expression and viral production, but has no effect on viral entry, replication, even polyprotein translation. Therefore, caspase-3 is exploited functionally by EV71 to facilitate its production, which suggests a novel therapeutic approach for the treatment and prevention of hand, foot, and mouth disease.

## Introduction

Hand, foot, and mouth disease (HFMD) is a febrile exanthematous disease prevalent in children younger than 5. The main symptoms of HFMD are vesicles on the palmar and plantar surfaces of the hands and feet, buccal mucosa, tongue, and buttocks. Severe clinical symptoms include acute flaccid paralysis, pulmonary edema, myocarditis, and encephalitis, sometimes leading to death (Chan et al., 2003). Unfortunately, drugs for HFMD therapy are not currently available, which may be largely because the pathogenic mechanism of HFMD has not been well elucidated.

Human enterovirus 71 (EV71) is a primary causative agent for HFMD and is associated with recent outbreaks in Asia (Gui et al., 2015; Li J. et al., 2016). EV71 belongs to the *Enterovirus* genus of the Picornaviridae family, which has a single-stranded, positive-sense RNA genome of about 7,400 bases consisting of 5′ and 3′ non-translated regions flanking a large open reading frame that encodes a polyprotein of about 2,193 amino acids. In host cells, this polyprotein is further cleaved into four structural (VP1–VP4) and seven non-structural (2A to 3D) proteins via the virus-encoded non-structural 2A and 3C proteases (Solomon et al., 2010). As a pathogenic mechanism of EV71, apoptosis has been studied in different cell lines (Chang et al., 2004; Shi et al., 2012; Lu et al., 2013), especially in regards to the activation of caspases, including caspase-8, caspase-9, and caspase-3, during viral replication (Chang et al., 2004). Caspase activation, which is central to the induction of apoptosis, involves a complex proteolytic cascade in which inactive proenzymes or zymogens are cleaved to generate active enzymes. The zymogens with longer prodomains, including caspase-8 and caspase-9, are activated first, and thus are called “initiator caspases,” while the zymogens with shorter prodomains, including caspase-3, subsequently activate DNase for DNA fragmentation, or active a host of cytoskeletal, cytosolic and nucleosomal proteins for cell death, and thus are called “executioner caspases” (Salvesen and Dixit, 1997; Villa et al., 1997; Hengartner, 2000; Larsen and Sorensen, 2017). Because caspase activation has the potential to disrupt virus replication, numerous viruses have adapted strategies to evade caspase activation (Henderson et al., 1993; Rao et al., 1997; Hardwick, 1998). Conversely, some viruses activate caspases, especially caspase-3, to facilitate their own replication (Wurzer et al., 2003; Martin et al., 2007; Richard and Tulasne, 2012). Unfortunately, the role of caspases in EV71 production is unknown, especially whether activated caspase-3 may be hijacked by EV71 to promote viral production or utilized by host cells to limit viral propagation. Viral non-structural protein of 3C is a protease with 183 amino acid, the catalytic triad of His40, Glu71, and Cys147 is important for the proteolytic activity, and each member of the catalytic triad is essential for the protease activity of EV71 3C protease (Lei et al., 2010; Cui et al., 2011; Li J. et al., 2017). The transient expression of the 3C protein encoded by EV71 has been reported to induce apoptosis by inducing caspase activation, while 3C mutant without proteolytic activity does not induce apoptosis (Li et al., 2002). And conversely, inhibition of caspase activity inhibits the proteolytic activity of 3C protease (Martin et al., 2007). However, the potential effect of inhibiting caspase-3 activity on EV71 viral propagation remains unclear. Additionally, the possibility that 3C may function by interacting directly with caspase-3 or other caspases has not been explored.

In this study, a specific peptide inhibitor of caspase-3 was used to determine the role of caspase-3 activation following EV71 virus infection. Our results show that caspase-3 inhibitor protects cells from the damage induced by EV71 infection and decreases the production of EV71 virions. We also demonstrate that the non-structural 3C protease encoded by EV71 binds to caspase-8 and caspase-9, but not caspase-3 and is responsible for caspase activation which depends on the proteolytic activity of 3C. Therefore, our findings suggest that caspase-3 is hijacked by EV71 for viral propagation.

## Materials and Methods

### Viruses and Cells

The Changchun077 strain of EV71 (Zhong et al., 2017) was propagated in human RD rhabdomyosarcoma cells (No CCL-136). RD and human HepG2 hepatocellular carcinoma (No HB-8065) cells were purchased from the ATCC (Manassas, VA, United States) and were maintained in Dulbecco’s modified Eagle’s medium (DMEM) (HyClone, Logan, UT, United States) supplemented with 10% fetal bovine serum (FBS) (GIBCO BRL, Grand Island, NY, United States). All the experiments involved in EV71 virus were done in a biosafety level 2 (BSL-2) laboratory.

### Plasmid Transfection

Plasmids pEGFP and pEGFP-3C were gifts from Dr. Jianwei Wang (Lei et al., 2012) for cell morphological observation and nuclear morphological study, and plasmids VR1012-HA and VR1012-3C-HA were prepared by our laboratory (Yu et al., 2015) for other studies, and the molecular weight of HA protein encoded by VR1012-HA was about 10 KD because the amino acid sequence was MYPYDVPDYAGARDYKDDDDKLQSPSSTRVIRYRGRSRPGP GSRSAVPSSCQPSVVCPSPVPSLTLEGATPTVLS which was HA (MYPYDVPDYA), flag (DYKDDDDK) and the sequence of VR1012. The 3C variant was constructed from VR1012-3C-HA by using a Site-Directed Mutagenesis Kit (Stratagene, La Jolla, CA, United States), and the C147G variant of 3C (3C Mutant) possessed a substitution of cysteine to glutamate at amino acid 147. According to the manufacturer’s protocol, 4 μg of plasmid were transfected together with 12 μl of Lipofectamine 2000 (Invitrogen) into HepG2 cells (RD cells are not easily transfected) in a 6-well plate for 36 h. Amounts of plasmid and Lipofectamine 2000 were increased for other experiments.

### Caspase Activity Assay

Caspase activity was analyzed using caspase-3/7 (Promega, G8090), caspase-8 (Promega, G8200), and caspase-9 (Promega, G8210) assays according to the manufacturer’s instructions. Briefly, cells were lysed using the manufacturer-provided homogeneous caspase reagent. The lysates were incubated at room temperature for 1.5 h before being read with a fluorometer at 485/530 nm. Luminescence values were detected by Fluoroskan Ascent FL (Thermo Scientific, MA, United States).

### Immunoprecipitation

HepG2 cells (5 × 10^5^ cells/well) were cultured in 10 cm dishes for 24 h and then were mock-transfected or transfected with 10 μg of VR1012-HA, VR1012-3C-HA, or VR1012-3C Mutant-HA plasmid for 36 h. Then the cells were harvested in IP-lysis buffer (50 mM Tris (pH 7.4), 150 mM NaCl, 1% NP-40, 0.25% sodium deoxycholate, 1 mM EDTA), and the lysates were added to Anti-HA affinity matrix (Roche) for immunoprecipitation of 3C-HA or HA protein. The bound proteins were analyzed by Western blotting.

### Caspase Inhibitor Treatment

Cells were pre-treated with 20 μM caspase-3 inhibitor (Z-DEVD-FMK, ApexBio, A1920), 20 μM caspase-8 inhibitor (Z-IETD-FMK, ApexBio, B3232), 20 μM caspase-9 inhibitor (Z-LEHD-FMK, ApexBio, B3233) or 0.05% DMSO in 10% DMEM as a control for 2 h and then washed with PBS. For experiments using HepG2 cells, the cells were transfected with 3C plasmid or a control vector for 2 h; for experiments using RD cells, the cells were infected with EV71 at a multiplicity of infection (MOI) of 1 or mock-treated for 2 h. Then, the cells were washed again with PBS and re-treated with 20 μM caspase inhibitor or 0.05% DMSO in 10% DMEM for the indicated times.

### Assessment of Cytopathic Effects

Cell morphological changes and nuclear morphological changes were observed to evaluate the cytopathic effects of 3C transfection or EV71 infection. The morphological changes were observed by microscopy (Olympus, Tokyo, Japan), and the nuclear changes were observed under a fluorescence microscope at an excitation wavelength of 350 nm with an emission filter at 460 nm (Leica, Nussloch, Germany) after staining with 5 mg/L Hoechst 33258 for 30 min.

### Determination of DNA Fragmentation by Agarose Gel Electrophoresis

Cells were trypsinized and both adherent and floating cells were collected by centrifugation at 1,000 ×*g* for 5 min. DNA fragmentation was assessed as previously described (Yu et al., 2007).

### Cell Counting Using a Hemocytometer

Trypan Blue (Sigma, St Louis, MO, United States) was used as a vital stain. Live cells appeared colorless and bright (refractile) under phase contrast, while dead cells stained blue and were non-refractile. After staining with a final concentration 0.2% trypan blue, live cells were visualized and counted using a hemocytometer.

### Enzyme-Linked Immunosorbent Assay (ELISA) for IL-6 Secretion

The culture media in different groups were examined for IL-6 using ELISA kits (Meiyan, Shanghai, China) according to the manufacturer’s instructions. Microplates were quantified using a microplate reader (Bio-Rad, Hercules, CA, United States). The absolute values of target proteins were calculated according to a standard curve.

### Cell Cycle Analysis by Flow Cytometry

Nuclear DNA content was measured using propidium iodide (PI) staining and fluorescence-activated cell sorting (FACS). Briefly, adherent cells were collected by treatment with trypsin and then washed with phosphate-buffered saline (PBS). Cells were fixed in 1 mL of cold 70% ethanol overnight at 4°C and resuspended in staining buffer (50 μg/mL PI [Sigma] and 20 μg/mL RNase in PBS) for 2 h at 4°C. The PI-stained cells were then analyzed by FACS (FACScan; BD), with at least 10,000 cells counted per sample. Data analysis was performed using ModFit LT, version 2.0 (Verity Software House) (Wang Z.Y. et al., 2017).

### Quantitative Real-Time RT PCR

Total RNA was extracted using Trizol reagent (Gibco-BRL, Rockville, MD, United States) as specified by the manufacturer. The RNA was treated with DNAse (DNase I-RNase-Free, Ambion) to remove any contaminating DNA. Then, 200 ng of RNA was reverse-transcribed with oligo dT primers using the High Capacity cDNA RT Kit (Applied Biosystems) in a 20 μL cDNA reaction as specified by the manufacturer. For quantitative PCR, the template cDNA was added to a 20 μL reaction with SYBR GREEN PCR Master Mix (Applied Biosystems) and 0.2 μM of primers for VP1, 5′UTR or GAPDH. The forward and reverse primer sequences for VP1 were 5′-AGCACCCACAGGCCAGAACACAC-3′ and 5′-ATCCCGCCCTACTGAAGAAACTA-3′, those for 5′UTR were 5′-CTTTGTGCGCCTGTTTTATAC-3′ and 5′-GGAAACAGAAGTGCTTGATCA-3′, and those for GAPDH were 5′-GCAAATTCCATGGCACCGT-3′ and 5′-TCGCCCCACTTGATTTTGG-3′. Amplification was carried out using an ABI Prism 7000 for 40 cycles with the following conditions: an initial denaturation at 95°C for 10 min; 40 cycles of 95°C for 15 s and 60°C for 1 min; then 1 cycle of 95°C for 1 min, 55°C for 30 s and 95°C for 30 s. Fold changes were calculated relative to GAPDH using the ΔΔCt method for gene-coding sequence analysis.

### 5′UTR Plasmid Construction and Luciferase Assays

The 5′UTR-luciferase plasmid was constructed as follows (Li Z. et al., 2016). The 5′UTR of EV71 was amplified using the following two pairs of forward and reverse primer 5′UTR primers: 5′-TCGAGTTAAAACAGCCTGTGGGTT-3′ (XhoI) and 5′-TGTTTAGCTGTGTTAAGGGT-3′ (HindIII); and 5′-GTTAAAACAGCCTGTGGGTT-3′ (XhoI) and 5′-AGCTTGTTTAGCTGTGTTAAGGGT-3′ (HindIII). The resulting products were inserted into the XhoI/HindIII sites of pGl3-Basic (Promega, Madison, WI, United States). HepG2 cells in 12-well plates were transfected with 1 μg 5′UTR-pGL3-Basic and 50 ng pRenilla for 4 h. Then 20 μM caspase-3 inhibitor was added for 20 h. At 24 h post-transfection, the luciferase activity was detected in cell extracts using Fluoroskan Ascent FL (Thermo Scientific, Waltham, MA, United States) with the Dual Luciferase Reporter Assay System (Promega), and the 5′UTR mRNA level was detected by quantitative real-time RT PCR.

### Virus Collection by Ultracentrifugation

For analysis of viral protein expression, supernatants were collected and brought up to 5 mL with DMEM. The supernatants were then subjected to repeated freezing and thawing for three cycles. Subsequently, the freeze/thawed preparations were centrifuged at 3,000 rpm for 20 min and the pellets were discarded. The medium with virus was then filtered with a 0.22 μm filter. Supernatants containing virus were centrifuged at 30,000 rpm for 3 h with 30% sucrose using a type 70.1 Ti rotor in a Beckman ultracentrifuge (Optimal L-100XP ultracentrifuge Beckman Coulter).

### Western Blot Analysis

Western blot analysis was performed as previously described (Yu et al., 2015). The following mouse or rabbit antibodies were used: anti-caspase-3 (Cell Signal, #9665), anti-caspase-8 (Cell Signal, #9746), anti-caspase-9 (Cell Signal, #9508), anti-histone (GenScript), anti-tubulin (Proteintech), and anti-VP1 polyclonal antibody (prepared by our laboratory). Mouse and rabbit secondary antibodies were obtained from Proteintech.

### Viral Titer Determination

Viral titers were determined by measuring the 50% tissue culture infective dose (TCID50) in a microtitration assay using RD cells, as described previously (Yu et al., 2015). Briefly, RD cells were seeded onto 96-well plates and incubated at 37°C for 24 h. Virus-containing supernatant was serially diluted 10-fold and 100 μL were added per well in octuplicate. The cytopathic effects were observed once per day until the experimental endpoint was reached. The TCID50 was determined by the Reed–Muench method (Reed, 1983), which is based on the assumption that 1 × 10^5^ TCID50/mL will produce 0.7 × 10^5^ plaque forming units/mL^1^.

### Statistical Analyses

Data are presented as means ± standard deviation (SD). Between-group differences were assessed using the Student’s *t*-test, and multiple-group differences were assed using *post hoc* test of one-way ANOVA in SPSS 10.0. ^∗^*P*-values of <0.05 were considered statistically significant.

## Results

### Non-structural 3C Protease of EV71 Binds Caspase-8 and Caspase-9 and Mediates Caspase-3 Activation, Which Mediates Its Cytopathic Effects

**FIGURE 1:**
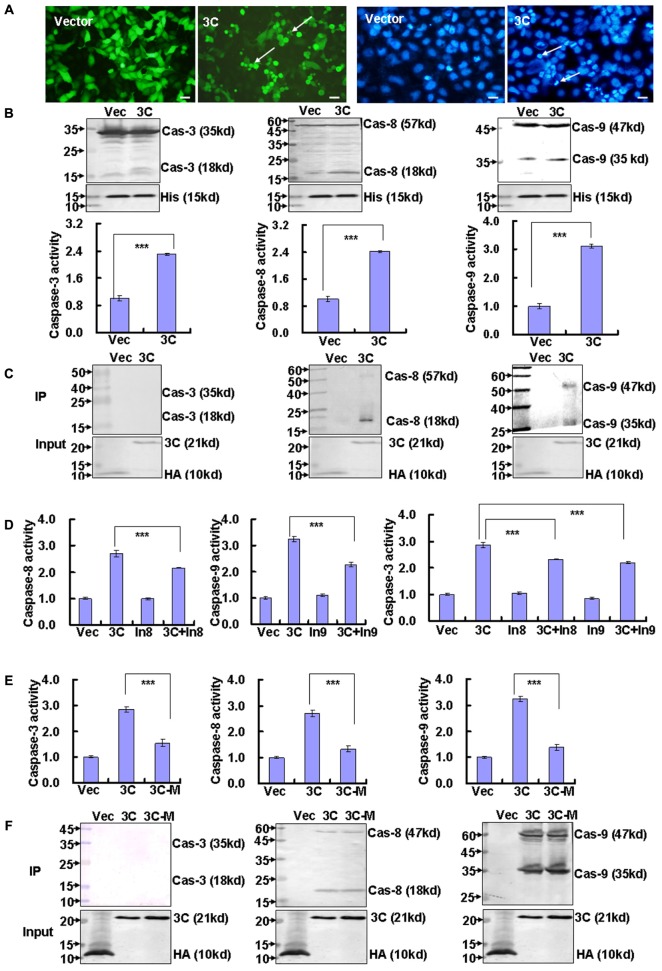
Non-structural 3C protease binds caspase-8 and caspase-9. **(A)** Cell morphologic analysis and nuclear morphologic analysis were performed in HepG2 cells because they are easy to transfect. Analyses were performed 36 h after transfection with 4 μg of pEGFP-3C (3C) or the corresponding control vector pEGFP (Vector). Bar = 20 μm. **(B)** The expression and activity of caspase-3, caspase-8, and caspase-9 proteins after transfection of HepG2 cells with VR1012-3C-HA or the corresponding control vector VR1012-HA (Vec) was assessed at 36 h by Western blot analysis (top) and by activity assay (lower). Histone is shown as a loading control. Luminescence values in each cell were calculated and normalized to 1.0 in vector-transfected cells. The results indicate the means ± SD of three independent experiments. ^∗∗∗^*P* < 0.001. **(C)** Immunoprecipitation of caspases with viral protein 3C. HepG2 cells were collected 36 h after transfection with 10 μg of VR1012-3C-HA (3C) or the corresponding control vector VR1012-HA (Vec). Anti-HA affinity matrix was used for immunoprecipitation of HA-tagged proteins, which were analyzed by Western blotting for caspase-3, caspase-8, caspase-9, and HA (for input). The results are representative of three independent experiments. **(D)** Caspase-8, caspase-9, and caspase-3 activity were analyzed by each activity assay after caspase-8 inhibitor and caspase-9 inhibitor treatment, respectively. Luminescence values in each group were calculated and normalized to 1.0 in VR1012-transfected and control-treated cells (Vec). The results indicate the means ± SD of three independent experiments. ^∗∗∗^*P* < 0.001. **(E)** Caspase-8, caspase-9, and caspase-3 activity were analyzed by each activity assay after 3C and 3C mutant transfection. Luminescence values in each group were calculated and normalized to 1.0 in VR1012-transfected cells (Vec). The results indicate the means ± SD of three independent experiments. ^∗∗∗^*P* < 0.001. **(F)** Immunoprecipitation of caspases with viral protein 3C or 3C mutant with loss of proteolytic activity. HepG2 cells were collected 36 h after transfection with 10 μg of VR1012-3C-HA (3C), VR1012-Cys147Glu-3C-HA (3C-M) or the corresponding control vector VR1012-HA (Vec). Anti-HA affinity matrix was used for immunoprecipitation of HA-tagged proteins, which were analyzed by Western blotting for caspase-3, caspase-8, caspase-9, and HA (for input). Cas-3, caspase-3; Cas-8, caspase-8; Cas-9, caspase-9; His, histone.

The non-structural 3C protease of EV71 is known to induce apoptosis of SF268 cells (Li et al., 2002), but the detailed mechanism is still unclear. We confirmed that 3C transfection promotes extensive cell rounding and detachment of HepG2 cells which is transfected more easily and also infected feasibly by EV71 infection (**Supplementary Figure S2**), as well as the appearance of condensed and rounded nuclei in 3C-transfected cells compared to control vector transfected cells (**Figure 1A**). To verify that the cytopathic effect of 3C is mediated by caspases, we analyzed the activation of caspase-3 (the executioner caspase), and caspase-8 and caspase-9 (the initiator caspases). Western blot and caspase activity analysis demonstrated that all three caspases were activated by 3C transfection (**Figure 1B**). To determine whether 3C interacts directly with these caspases, we performed immunoprecipitation assays. Our results suggested that 3C binds caspase-8 and caspase-9, but not caspase-3 (**Figure 1C**). To confirm whether 3C-induced caspase-3 activation and cytopathy is directly associated with activation of caspase-8 or caspase-9, we inhibited the activity of caspase-8 and caspase-9 with each inhibitor. And it is confirmed that caspase-3 activity is inhibited after inhibiting caspase-8 or caspase-9 activity in 3C-transfected cells (**Figure 1D**). Meanwhile non-structural 3C of EV71 is a protease (Li et al., 2002), but it is not clear about the relationship of proteolytic activity and activating caspases of 3C, so the effect of proteolytic activity of 3C on the activation of caspases is determined through detecting the activity of caspase-3, caspase-8, and caspase-9 after transfection of 3C or 3C mutant without protease ability, and it is confirmed that compared to 3C, 3C mutant decreases the activity of caspase-3, caspase-8, and caspase-9, respectively (**Figure 1E**), but 3C mutant possesses the ability of binding caspase-8 and caspase-9 as well as 3C (**Figure 1F**). Therefore, these results are consistent with the possibility that 3C binds caspase-8 and caspase-9 to activate them, which subsequently to activate caspase-3, and the activation of them depends on the proteolytic ability of 3C.

**FIGURE 2:**
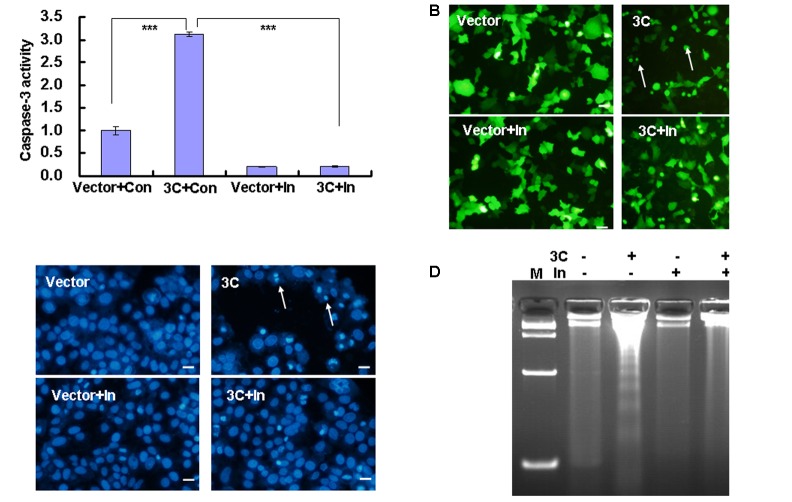
Caspase-3 inhibitor inhibits caspase-3 activation and cytopathy in 3C-transfected cells. **(A)** Caspase-3 activity was analyzed by caspase-3/7 activity assay. Luminescence values in each group were calculated and normalized to 1.0 in VR1012-transfected and control-treated cells. The results indicate the means ± SD of three independent experiments. ^∗∗∗^*P* < 0.001. **(B)** Cell morphologic analysis and **(C)** nuclear morphologic analysis of HepG2 cells were performed 36 h after transfection with 4 μg of pEGFP-3C (3C) or the corresponding control vector pEGFP (Vector) with/without caspase-3 inhibitor. Bar = 20 μm. **(D)** DNA fragmentation was analyzed by agarose gel electrophoresis. M indicates marker. Data are representative of three individual experiments (*n* = 3). Con, 0.05% DMSO in 10% DMEM; In, caspase-3 inhibitor.

3C-induced apoptosis is prevented by caspase inhibitors (Li et al., 2002), which further raises the possibility that caspase-3 may be targeted by 3C to induce cytopathy. As expected, the caspase-3 inhibitor Z-DEVD-FMK decreased the activity of caspase-3 in 3C-transfected cells (**Figure 2A**). Furthermore, consistent with the latter findings (Li et al., 2002), Z-DEVD-FMK inhibited cell rounding and detachment (**Figure 2B**), as well as the appearance of condensed and rounded nuclei in 3C-transfected HepG2 cells (**Figure 2C**). Caspase inhibitor also inhibited the DNA fragmentation induced by 3C transfection (**Figure 2D**), which further supports its potential role in 3C-induced apoptosis in transfected HepG2 cells.

### Caspase-3 Inhibitor Protects RD Cells From Cytopathic Effects Induced by EV71 Infection

**FIGURE 3:**
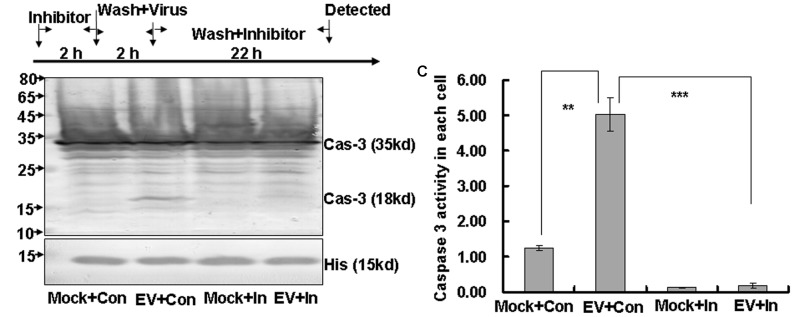
Caspase-3 inhibitor inhibits caspase-3 activation in EV71 infected cells. **(A)** Flow diagram of RD cell treatment with caspase-3 inhibitor. RD cells were pre-treated with 20 μM caspase-3 inhibitor (Inhibitor) or 0.05% DMSO in 10% DMEM (Con) for 2 h. The cells were then washed with PBS and mock-infected or infected with EV71 at an MOI of 1. After 2 h, the cells were washed again with PBS and re-treated with 20 μM caspase-3 inhibitor or 0.05% DMSO in 10% DMEM (Con) for another 22 h. **(B)** Caspase-3 expression was analyzed by Western blotting. Histone is shown as a loading control. The results are representative of three independent experiments. **(C)** Caspase-3 activity was analyzed by caspase3/7 activity assay kits. Luminescence values were detected and cell numbers were calculated for standardization on a per cell basis. Mock, mock-infected; Con, 0.05% DMSO in 10% DMEM; EV, EV71 infection; In, caspase-3 inhibitor; Cas-3, caspase-3; His, histone. The results indicate the means ± SD of three independent experiments. ^∗∗^*P* < 0.01, ^∗∗∗^*P* < 0.001.

To further confirm the effect of caspase-3 on apoptosis induced by EV71 infection (Chang et al., 2004; Shi et al., 2012; Lu et al., 2013), we used caspase inhibitor to assess the role of caspase-3 activation in apoptosis and viral propagation. RD cells were pre-treated with different doses of Z-DEVD-FMK or 0.05% DMSO in 10% DMEM for 2 h and then were washed with PBS and mock-infected or infected with EV71 at an MOI of 1. Two hours later the cells were washed again and re-treated with a corresponding dose of Z-DEVD-FMK or 0.05% DMSO in 10% DMEM for another 22 h (**Figure 3A**). Doses of caspase-3 inhibitor of 20 μM or less had no obvious toxicity, but doses of greater than 20 μM significantly decreased the cell survival (*P* < 0.001, **Supplementary Figure S1A**). Furthermore, 20 μM Z-DEVD-FMK provided the best protective effect on cell survival after EV1 infection (**Supplementary Figure S1B**). Therefore, we used 20 μM caspase-3 inhibitor in subsequent experiments.

Western blot analysis suggested that the active caspase-3 expression was increased at 24 h post-infection with EV71, but that the addition of caspase-3 inhibitor blocked this increase (**Figure 3B**). To confirm the effects of caspase-3 inhibitor on caspase-3 activation, we also performed caspase activity assays. The results verify that EV71 infection increased caspase-3 activity (EV+Con; 5.02 ± 0.47 RLU) compared to the mock-infected group (Mock+Con; 1.25 ± 0.07 RLU; *P* < 0.01), but that caspase-3 inhibitor treatment reversed this activation (EV+In versus EV+ Con; *P* < 0.001) (**Figure 3C**). Therefore, caspase-3 inhibitor effectively inhibits caspase-3 activity in EV71-infected RD cells.

**FIGURE 4:**
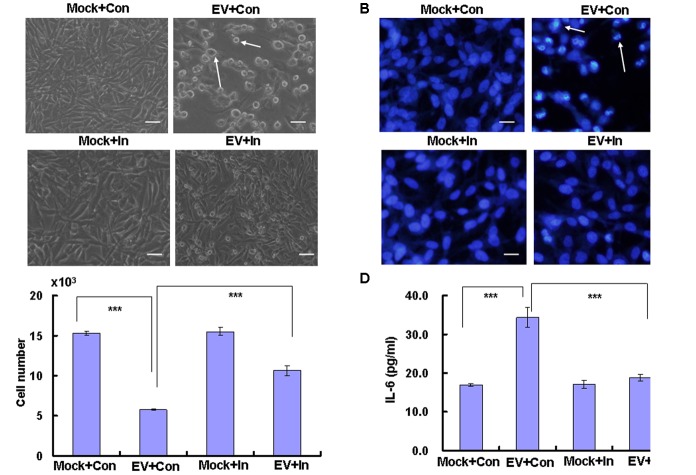
EV71-induced cytopathic effects are blocked by caspase-3 inhibitor. RD cells were treated with 20 μM of caspase-3 inhibitor (In) or 0.05% DMSO in 10% DMEM (Con) for 2 h, and then were mock-infected (Mock) or infected with EV71 (EV) at an MOI of 1. After 2 h, the cells were re-treated with caspase-3 inhibitor (In) or 0.05% DMSO in 10% DMEM (Con) for another 22 h. **(A)** Morphologic analysis of the effect of caspase-3 inhibitor on cell death after EV71 infection. Cell morphology was visualized by light microscopy. Arrows indicate dead cells. Bar = 20 μm. The results are representative of three independent experiments. **(B)** Nuclear morphologic analysis of the effect of caspase-3 inhibitor on cell death after EV71 infection. The nuclear morphology was visualized by light microscopy after Hoechst 33258 staining. Arrows indicate dead cells. Bar = 10 μm. The results are representative of three independent experiments. **(C)** Cell number analysis of the effect of caspase-3 inhibitor on cell death after EV71 infection. The cell numbers were counted after trypan blue staining. **(D)** Effect of caspase-3 inhibitor on the release of inflammatory cytokine. The level of IL-6 in cultural medium was assessed by ELISA. The results show the means ± SD of three independent experiments. ^∗∗∗^*P* < 0.001.

Viral invasion may cause cytopathic effects, which are characterized by structural changes that are necessary for efficient virus replication but occur at the expense of the host cell (Holmes, 1975). To determine whether caspase-3 modulates the cytopathic effect caused by EV71 virus, we examined the morphological changes associated with EV71 infection. Our results demonstrate that numerous cells were rounded up and detached from the bottom of the dish at 24 h post infection, representing the typical cytopathic effect of EV71, however, caspase-3 inhibitor reduced the detached cells after EV71 infection (**Figure 4A**). Furthermore, EV71-infected cells exhibited condensed chromatin, which is another hallmark of cytopathy, whereas caspase-3 inhibitor blocked the appearance of condensed and round nuclei after EV71 infection (**Figure 4B**). The cell number was also decreased by EV71 infection (0.57 ± 0.01 × 10^4^) compared to mock infection (1.53 ± 0.03 × 10^4^) (*P* < 0.001), whereas caspase-3 inhibitor increased the cell number after EV71 infection (1.06 ± 0.06 × 10^4^) compared to EV71 infection alone (**Figure 4C**). Therefore, caspase-3 inhibitor blocks the cytopathic effect of EV71 and protects host cells from damage induced by EV71 infection.

### Caspase-3 Inhibitor Decreases the Level of IL-6 in Culture Medium From RD Cells Infected With EV71

EV71 infection leads to the release of inflammatory cytokines, which are associated with the clinical symptoms of HFMD (Lin et al., 2003; Zhang et al., 2017). To determine the effect of caspase-3 inhibitor on the release of the inflammatory cytokine IL-6, ELISA was performed. The content of IL-6 in the culture medium was increased by EV71 infection compared to mock infection (*P* < 0.001), while caspase-3 inhibitor reversed the increase (*P* < 0.001) (**Figure 4D**). Therefore caspase-3 inhibitor inhibits the secretion of IL-6.

### Caspase-3 Inhibitor Interferes With the Cell Cycle Status That Is Favored by EV71

**FIGURE 5:**
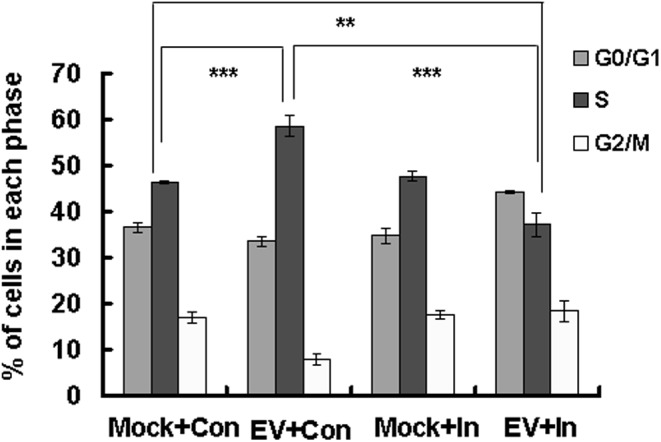
Effect of caspase-3 inhibitor on the cell cycle status favored for EV71 viral production. RD cells were treated with 20 μM of caspase-3 inhibitor (In) or 0.05% DMSO in 10% DMEM (Con) for 2 h, and then mock-infected (Mock) or infected with EV71 (EV) at an MOI of 1. After 2 h, the cells were re-treated with caspase-3 inhibitor (In) or 0.05% DMSO in 10% DMEM (Con) for an additional 22 h. Cell-cycle profiles were determined by flow cytometry. Histograms show the percentage of cells in each phase of the cell cycle as analyzed by the ModFit LT program. The results indicate the means ± SD of three independent experiments. ^∗∗^*P* < 0.01, ^∗∗∗^*P* < 0.001.

EV71 is known to induce S phase arrest to facilitate its own production (Yu et al., 2015). To determine whether caspase-3 inhibitor influences this S phase arrest, we performed FACS analysis. As expected, the percentage of S phase cells was higher for the EV71 infection group (58.52 ± 2.29) than for the control group (46.37 ± 0.26; *P* < 0.001). However, the addition of caspase-3 inhibitor decreased the percentage of cells in S phase (37.25 ± 2.58; *P* < 0.001), which was lower than in the mock infected or control treated group (*P* < 0.01) (**Figure 5**). These results suggest that caspase-3 inhibitor reverses the S phase arrest that is mediated by EV71 infection, and raising the possibility that caspase-3 might directly participate in cell cycle regulation.

### Caspase-3 Inhibitor Inhibits Viral Protein Expression and Decreases the Infectious Virus Production of EV71

**FIGURE 6:**
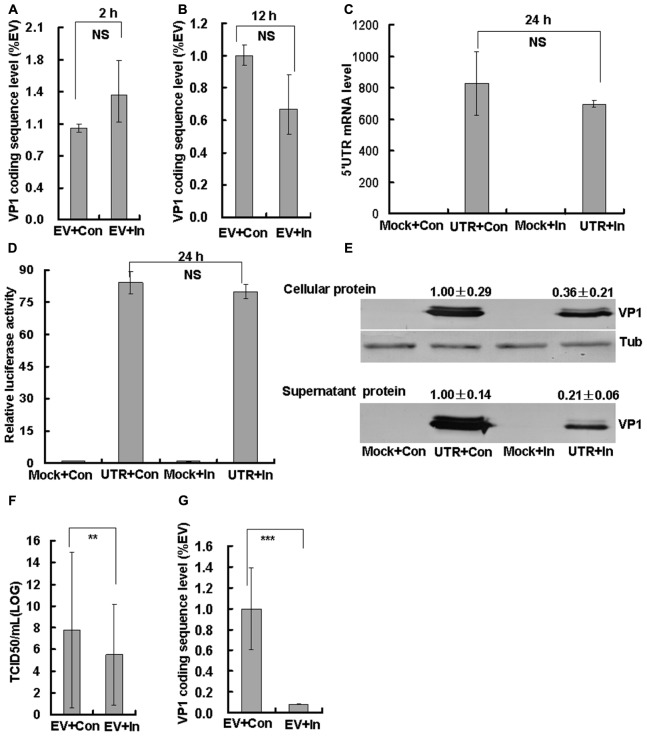
Effect of caspase-3 inhibitor on EV71 viral production. RD cells were treated with 20 μM of caspase-3 inhibitor (In) or 0.05% DMSO in 10% DMEM (Con) for 2 h. Then the cells were mock-infected (Mock) or infected with EV71 (EV) at an MOI of 1. After 2 h, the cells were re-treated with caspase-3 inhibitor (In) or 0.05% DMSO in 10% DMEM (Con) for the indicated times. At 2 h **(A)** and 12 h **(B)** post-infection, intracellular EV71 RNA levels were detected in control or caspase-3 inhibitor-treated RD cells by real-time quantitative PCR. The results are standardized to GAPDH mRNA as a control and normalized to 1.0 in EV-infected cells. NS: No significant difference. **(C,D)** HepG2 cells in 12-well plates were transfected with 1 μg 5′UTR-pGL3-Basic and 50 ng pRenilla for 4 h. Then, 20 μM caspase-3 inhibitor or control medium was added for 20 h. At 24 h post-transfection, the 5′UTR mRNA level **(C)** was detected and standardized using GAPDH mRNA as a control and normalized to 1.0 in mock-transfected cells (transfected with pgl3-basic). Luciferase activity **(D)** was detected and normalized to 1.0 in mock-transfected cells. **(E)** VP1 expression in cellular lysates or in viral supernatants was determined after growth in control medium or caspase-3 inhibitor medium at 24 h post-infection. The numerical value shown is the ratio of VP1 protein intensity to the intensity in the caspase-3 inhibitor treatment group. The results are representative of three independent experiments. Tub: Tubulin. **(F)** Total progeny viruses (supernatant and cellular viruses) were titrated using RD cells at 24 h post-infection. Quantitative analysis of the TCID50/mL is detected and log10^TCID50/mL^ is shown. The results indicate the means ± SD of three independent experiments. ^∗∗^*P* < 0.01. **(G)** At 24 h post-infection, supernatant EV71 RNA levels were detected in control or caspase-3 inhibitor-treated RD cells by real-time quantitative PCR. The results are standardized to GAPDH mRNA as a control and normalized to 1.0 in EV-infected and control-treated cells. The results indicate the means ± SD of three independent experiments. ^∗∗∗^*P* < 0.001.

To evaluate whether caspase-3 inhibitor inhibits viral production, we examined viral entry, viral replication, viral protein expression, and virulence of EV71 after infection and treatment of RD cells with caspase-3 inhibitor. First, the EV71 viral genomic level was assessed by real time PCR of the VP1 coding sequence. Caspase-3 inhibitor did not affect the viral genomic level at 2 h post infection (viral entry phase; **Figure 6A**) or at 12 h post-infection (viral replication; **Figure 6B**). As a measurement of viral translation, a 5′UTR-pGl3-Basic luciferase plasmid was constructed and transfected into HepG2 cells. Caspase-3 inhibitor did not affect the 5′UTR mRNA level (**Figure 6C**) or the luciferase activity of transfected cells (**Figure 6D**) at 24 h post-transfection, which further verifies that caspase-3 inhibitor does not affect viral translation. However, caspase-3 inhibitor decreased both the intracellular VP1 expression from 1.00 ± 0.29 to 0.36 ± 0.21 (by 2.78-fold, *P* < 0.05), and the supernatant VP1 expression from 1.00 ± 0.14 to 0.21 ± 0.06 (by 4.76-fold, *P* < 0.001) at 24 h post-infection as assessed by Western blot assays (**Figure 6E**). Therefore Caspase-3 inhibitor did not affect viral genome replication and polyprotein translation, but decreased the viral protein expression in cells. Finally, we analyzed EV71 production at 24 h post-infection. The TCID50/mL of EV71-infected cells (592.00 ± 148.23 × 10^5^), was dramatically reduced by the addition of caspase-3 inhibitor (3.33 ± 0.44 × 10^5^) (about 178-fold, **Figure 6F**). To further analyze the reason of decreased TCID50/mL by caspase-3 inhibitor, the supernatant viruses after caspase-3 inhibitor treatment were collected for real-time PCR analysis, and it was confirmed that the viral genomic level was decreased from 1.00 ± 0.39 to 0.08 ± 0.003 (by 12.20-fold, *P* < 0.001, **Figure 6G**), which indicated that decreasing packaging of viral genomes into capsids by caspase-3 inhibitor partly explained the decreased TCID50/mL after caspase-3 inhibitor treatment. Therefore, caspase-3 inhibitor does not affect viral entry and viral genome replication, but decreases viral protein expression and production.

## Discussion

EV71 is the primary causative agent for HFMD outbreaks in Asian countries (Liu et al., 2011; Wang et al., 2012). Unfortunately, currently available antiviral drugs have limited efficacy against EV71 infection, though inhibitors targeting various phases of the EV71 lifecycle (Wang L. et al., 2015) are currently at the preclinical stage. Therefore, we reasoned that an advanced understanding of the pathogenic mechanism of EV71 virus may help to provide a basis for the design of effective drugs to treat HFMD.

Previous studies have shown that the viral non-structural protein 3C induces cell death (Li B. et al., 2017), and that inhibiting caspase activity can block the cell death induced by 3C. To further explore the mechanism of 3C, we performed Western blot analysis and immunoprecipitation assays in hepatocellular carcinoma (HepG2) cells. Our results demonstrated that 3C activated caspase-3, caspase-8, and caspase-9. Inhibiting the activity of caspase-8 and caspase-9, the activity of caspase-3 was also inhibited in 3C-tranfected cells. 3C directly bound caspase-8 and caspase-9, but not caspase-3. Therefore 3C binds caspase-8 and caspase-9 to activate them, and then activates caspase-3. As a protease, the catalytic triad of His40, Glu71 and Cys147 of 3C is important for the proteolytic activity, and each member of the catalytic triad is essential for the protease activity of EV71 3C protease (Lei et al., 2010; Cui et al., 2011; Li J. et al., 2017), and the study confirmed that Cys147Glu of 3C did not activate caspase-8, caspase-9, and caspase-3 although still bound with caspase-8 and caspase-9. Therefore that 3C activates caspases depends on the proteolytic ability of 3C. Furthermore, caspase-3 inhibitor decreased the activity of caspase-3 to inhibit cell apoptosis-induced by 3C. Therefore, our results are consistent with the possibility that 3C activates caspase-3 to induce apoptosis through the activation and binding of caspase-8 and caspase-9. Interestingly, in some neural cell lines, caspase-9 is activated to induce cell apoptosis by EV71 infection, but caspase-8 is not activated (Chang et al., 2004; Wang C. et al., 2015). Therefore, we speculate that in some cell lines a specific cellular factor may inhibit 3C from binding caspase-8 or potentially from activating it. Moreover, our previous study proves that 3C also has the ability to manipulate host cell cycle (Zhong et al., 2017), although it is not clear about the detail reason of cell cycle arrest, it is speculated that 3C might firstly induce DNA damage on the fact that activated caspase-3 activates DNase to cut DNA (Larsen and Sorensen, 2017), then lead to cell cycle arrest for repairing DNA damage, while finally cell death due to damage beyond repair. Therefore it is concluded that 3C is a multifunctional protein which is involved in the cell cycle arrest, apoptosis and hydrolysis, and 3C helps viral proliferation possibly through creating favorable environment or directly participating viral maturation.

EV71 virus has been demonstrated previously to activate caspase-3 to induce apoptosis of host cells (Chang et al., 2004; Shi et al., 2012; Lu et al., 2013), but until now, the role of activated caspase-3 in the pathogenic mechanism of EV71 virus was unclear. In this study, we used the caspase-3 inhibitor Z-DEVD-FMK to assess the role of caspase-3 activation on viral function. Western blotting and caspase-3 activity assays verified the function of Z-DEVD-FMK in inhibiting caspase-3 activity. Furthermore, caspase-3 inhibitor attenuated the cytopathic effects of EV71. Cell death is known to lead to inflammatory responses caused by the release of cellular contents, which has been demonstrated by *in vivo* and *in vitro* experiments (Chang et al., 2004; Wang et al., 2004; Zeng et al., 2013). In this study, we confirmed that caspase-3 inhibitor decreased the level of the extracellular inflammatory factor IL-6, as well it was found by Wang that caspase-3 inhibitor up-regulated the interferon response to increase the anti-viral ability of interferon (Wang C. et al., 2017), which indicated that caspase-3 inhibition might be directly involved in inflammation response, in addition to inhibiting inflammation factor release through inhibiting cell death. Cell death is also known to lead to the loss of function of damaged cells. For example, neural cell death may lead to amnesia or mental disorder (Wu et al., 2015), and muscle cell death may lead to paralysis or myasthenia (Burr and Molkentin, 2015). On the basis of our findings, caspase-3 inhibitor maintains host function by preventing the occurrence of the inflammatory responses or other detrimental events associated with cytopathic cell death caused by EV71 infection.

In our previous study, we also found that EV71 manipulates cell cycle arrest at S phase through non-structural protein 3D, and that in turn, S phase-synchronization promotes EV71 viral production (Yu et al., 2015). In this study, we determined that caspase-3 inhibitor treatment prevents S phase arrest and viral production. Therefore, the manipulation of the host cell status by caspase-3 may provide a mechanism by which EV71 increases its propagation. Interestingly, the percentage of cells in S phase for EV71-infected and caspase-3 inhibitor-treated cells was found to be lower than that observed in mock-infected and control-treated cells. Although the mechanism is not clear, we speculate that non-structural protein 3D may promote S phase arrest in the presence of caspase-3 activation, but G0/G1 phase arrest in the absence of caspase-3 activation. Another possibility is that in the presence of caspase-3 activation, non-structural 3D is responsible for S phase arrest, and in the absence of caspase-3 activation, other viral proteins are responsible for G0/G1 arrest. Further investigation is needed to distinguish these possibilities. Therefore, caspase-3 activation is very important for S phase arrest induced by EV71 infection, and caspase-3 might directly participate in cell cycle control.

To determine whether caspase-3 inhibitor prevents EV71 viral replication or other steps within the process of virus production, we performed a series of analyses of VP1 genome levels, protein expression and virulence. Our results demonstrated that caspase-3 inhibitor did not affect viral entry, viral genome replication or polyprotein translation; however, caspase-3 inhibitor significantly blocked intracellular viral protein expression (about 2.78-fold) and supernatant viral protein expression (about 4.76-fold), decreased supernatant viral genome level (about 12.20-fold) and decreased EV71 production (about 178-fold). On the basis of these findings, caspase-3 is likely to be activated by EV71 for viral assembly and maturation.

Collectively, our findings further improve our understanding of the EV71 viral pathogenic mechanisms and identify caspase-3 inhibition as a potential strategy for the treatment and prevention of HFMD.

## Author Contributions

JY designed the experiments and wrote the paper. FS, XY, TZ, ZW, XM, and XL conducted the experiments. ZL, SZ, WH, YZ, and WZ prepared the virus, cell, and regents.

## Conflict of Interest Statement

The authors declare that the research was conducted in the absence of any commercial or financial relationships that could be construed as a potential conflict of interest.

## Footnotes

1 www.protocol-online.org/biology-forums/posts/1664.html

## Supplementary Material

FIGURE S1 The effect of caspase-3 inhibitor on cell growth and the protective effect of caspase-3 inhibitor on EV71 infected cells. **(A)** The cell activity was detected by MTT analysis after 0 μM, 5 μM, 10 μM, 20 μM, 40 μM, and 80 μM of caspase-3 inhibitor treatment at 24 h in RD cells. **(B)** RD cells were treated with 0 μM, 5 μM, 10 μM, 20 μM, 40 μM, and 80 μM of caspase-3 inhibitor for 2 h. Then the cells were infected with EV71 at an MOI of 1. After 2 h, the cells were re-treated with caspase-3 inhibitor. At 24 h post-infection, the cell activity was detected by MTT analysis. The results show the means ± SD of three independent experiments. ^∗∗∗^*P* < 0.001.

FIGURE S2 EV71-induced cytopathic effects are blocked by caspase-3 inhibitor in HepG2 cells. HepG2 cells were treated with 20 μM of caspase-3 inhibitor (In) or 0.05% DMSO in 10% DMEM (Con) for 2 h, and then were mock-infected (Mock) or infected with EV71 (EV) at an MOI of 5. After 2 h, the cells were re-treated with caspase-3 inhibitor (In) or 0.05% DMSO in 10% DMEM (Con) for another 22 h. **(A)** Morphologic analysis of the effect of caspase-3 inhibitor on cell death after EV71 infection. Cell morphology was visualized by light microscopy. Arrows indicate dead cells. Bar = 20 μm. The results are representative of three independent experiments. **(B)** Nuclear morphologic analysis of the effect of caspase-3 inhibitor on cell death after EV71 infection. The nuclear morphology was visualized by light microscopy after Hoechst 33258 staining. Arrows indicate dead cells. Bar = 10 μm. The results are representative of three independent experiments.
