# Supplementary figures and images for: Caspase-3 Inhibition Attenuates the Cytopathic Effects of EV71 Infection
Source: Front Microbiol. 2018 Apr 26;9:817. doi: 10.3389/fmicb.2018.00817 (PMC5932146; doi:10.3389/fmicb.2018.00817)

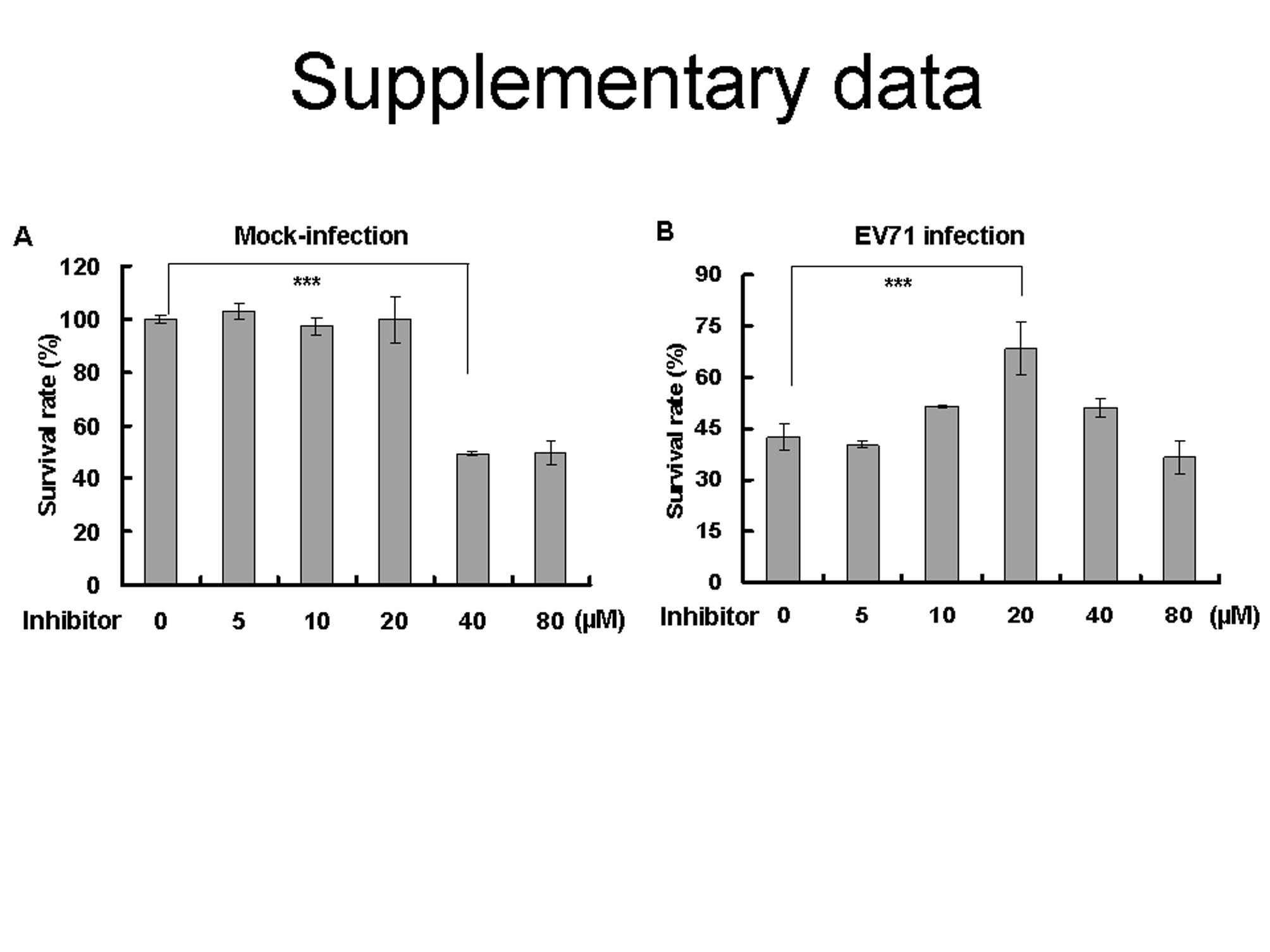

Supplement: FIGURE S1 — The effect of caspase-3 inhibitor on cell growth and the protective effect of caspase-3 inhibitor on EV71 infected cells. (A) The cell activity was detected by MTT analysis after 0 μM, 5 μM, 10 μM, 20 μM, 40 μM, and 80 μM of caspase-3 inhibitor treatment at 24 h in RD cells. (B) RD cells were treated with 0 μM, 5 μM, 10 μM, 20 μM, 40 μM, and 80 μM of caspase-3 inhibitor for 2 h. Then the cells were infected with EV71 at an MOI of 1. After 2 h, the cells were re-treated with caspase-3 inhibitor. At 24 h post-infection, the cell activity was detected by MTT analysis. The results show the means ± SD of three independent experiments. ∗∗∗P < 0.001. [file Image_1.TIF]

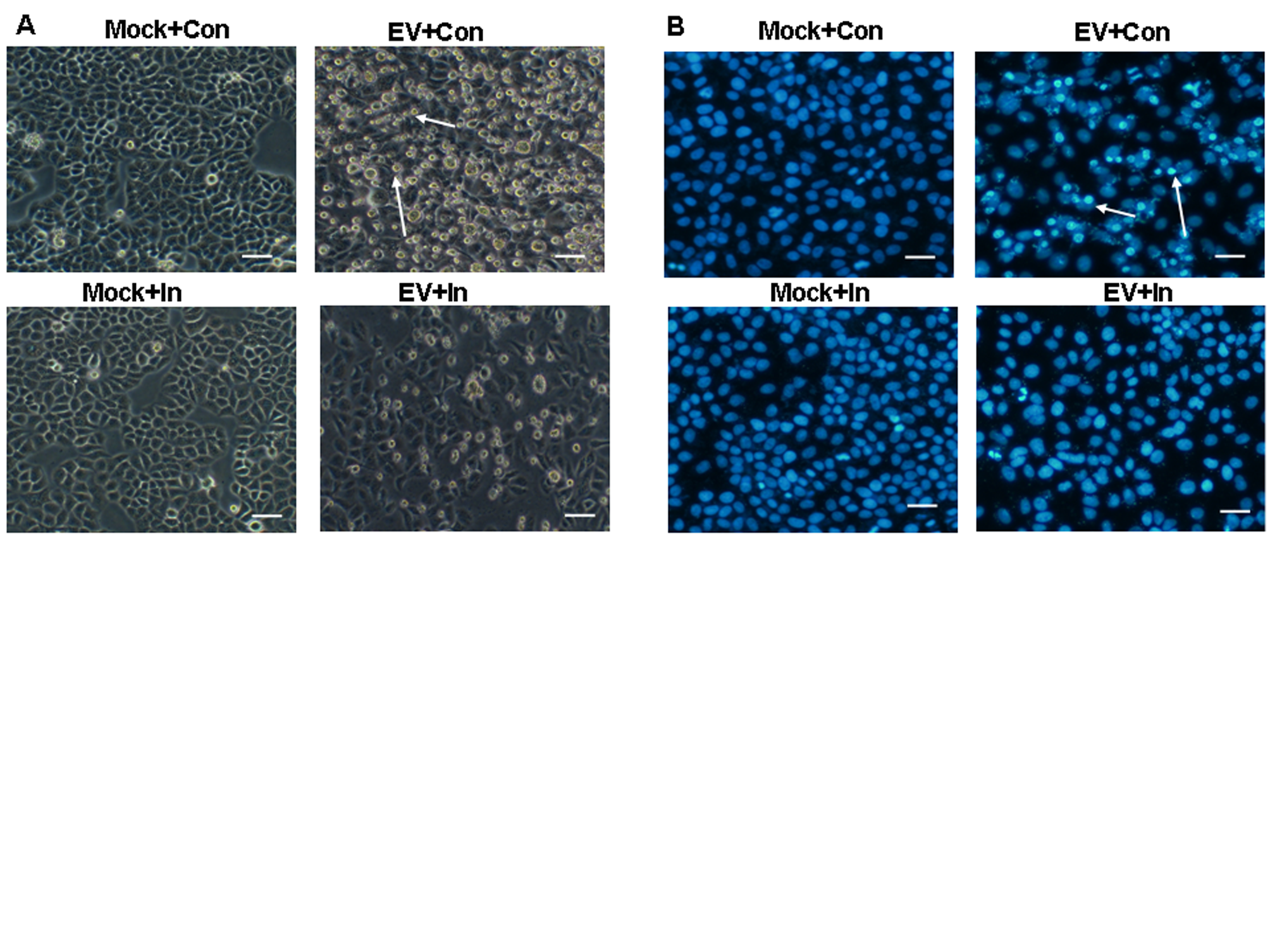

Supplement: FIGURE S2 — EV71-induced cytopathic effects are blocked by caspase-3 inhibitor in HepG2 cells. HepG2 cells were treated with 20 μM of caspase-3 inhibitor (In) or 0.05% DMSO in 10% DMEM (Con) for 2 h, and then were mock-infected (Mock) or infected with EV71 (EV) at an MOI of 5. After 2 h, the cells were re-treated with caspase-3 inhibitor (In) or 0.05% DMSO in 10% DMEM (Con) for another 22 h. (A) Morphologic analysis of the effect of caspase-3 inhibitor on cell death after EV71 infection. Cell morphology was visualized by light microscopy. Arrows indicate dead cells. Bar = 20 μm. The results are representative of three independent experiments. (B) Nuclear morphologic analysis of the effect of caspase-3 inhibitor on cell death after EV71 infection. The nuclear morphology was visualized by light microscopy after Hoechst 33258 staining. Arrows indicate dead cells. Bar = 10 μm. The results are representative of three independent experiments. [file Image_2.TIF]
